# The impact of regional sports industry aggregation on residents’ health level in China

**DOI:** 10.1038/s41598-024-60564-y

**Published:** 2024-05-13

**Authors:** Min Luo, Lingming Chen

**Affiliations:** 1 School of Physical Education, Xinyu University (XYU), Xinyu, 338004 China; 2 School of Economics and Management, Xinyu University (XYU), Xinyu, 338004 China

**Keywords:** Sports, Sports industry aggregation, Residents’ health, Spatial spillover, China, Physiology, Health care

## Abstract

Health is the basis for human survival and development and is an important symbol to evaluate a country's economic growth and social progress. This article measures the degree of sports industry agglomeration in different regions of China and uses the Moran index to verify the existence of global autocorrelation in sports agglomeration. Next, the spatial Durbin model was used to verify the spatial spillover effect of sports industry agglomeration on the health level of residents, and the following conclusions were obtained.Firstly, there is spatial autocorrelation and heterogeneity in the clustering level of China's sports industry. The spatial distribution is extremely uneven, and different regions have formed relatively stable spatial patterns. Secondly, the degree of aggregation of the sports industry can reduce the number of per capita visits and have a positive spatial spillover effect on the health of residents. Not only can it promote the health level of residents in the province, but it also has spatial spillover effects on surrounding areas.Finally, based on the research results, the following conclusions are proposed in this article. Policy recommendations include increasing investment in sports talent cultivation, accelerating the construction of sports center cities, and increasing residents' attention to sports to improve residents' health.

## Introduction

With the deepening of globalization and rapid socio-economic development, the sports industry, as an important socio-economic activity, is increasingly valued in the development strategies of various countries. The sports industry is an economic activity and an essential, meaningful way to reflect social culture and promote people's health. The agglomeration degree of the sports industry, as a critical indicator to measure the development level of the regional sports industry, has attracted widespread attention for its impact on the health level of residents. The agglomeration of the sports industry not only means the degree of aggregation of related industries in a particular region but also reflects the enthusiasm and competitiveness of the region in the development of the sports industry. At the same time, the agglomeration of the sports industry also means that residents in this region may have richer resources and conditions regarding sports exercise, health awareness, and other aspects.

At the same time, the health level of the population, as one of the critical indicators for measuring the social development level of a country or region, is directly related to the economic development, social stability, and people's quality of life of the country or region. Especially in China, with its vast territory, diverse culture, and uneven economic development, there are significant differences in the agglomeration degree of the sports industry and the health level of the population in different regions. Some developed areas have advanced sports facilities, abundant sports resources, and an active sports atmosphere, and their residents have relatively more opportunities for sports exercise and healthier lifestyles. However, in some underdeveloped areas, due to economic conditions, cultural traditions, and other factors, the development of the sports industry is relatively lagging, and the health level of residents is also relatively low.

Therefore, in-depth research on the relationship between regional sports industry agglomeration and population health level not only helps to reveal the positive impact mechanism of the sports industry on residents' health but also provides targeted policy recommendations for the development and health promotion of the sports industry in different regions, promotes the vigorous growth of the sports industry in various regions, improves the health level of the whole population, and achieves the strategic goal of comprehensively building socialist modernization in the country. Compared with previous research, the innovation of this article is to explore the relationship between sports industry agglomeration and resident health in a broader context, exploring the development status of the sports industry, the overall level of resident health, and the interaction between the two; Secondly, traditional models usually only consider the impact of sports facilities on the health level of residents, while ignoring spatial spillover effects. The spatial Durbin model can help us explore the indirect impact of sports industry clustering on the health of residents in other regions, thereby more comprehensively evaluating its effect on improving the overall health level of the region.

## Literature review

The development of the sports industry is relatively late and has not yet formed a scale. There is not much research on the sports industry in academic circles. General research mainly focuses on the impact of the sports industry on economic-related characteristics, measuring the agglomeration of the sports industry, and studying the impact on residents' health levels.

Firstly, regarding the calculation of the aggregation degree of the sports industry. The difference between the existing literature on measuring sports industry agglomeration mainly lies in measurement methods and the research object. Song ^1^ uses the spatial agglomeration index to measure the industrial agglomeration degree of China's sports products manufacturing industry and related sub-industries and discusses the development of sports industry clusters. The research of Wang Liangjian et al.^2^ used many methods to estimate the aggregation degree of the sports industry, including information entropy, industry concentration degree, location Gini coefficient, spatial separation index, location quotient, and kernel density estimation. The research of Maowei et al.^3^ takes Beijing and Guangzhou sports industry agglomeration as the object, mainly from the perspective of comparative advantage theory and new economic geography theory, to explore the dynamics and action mechanisms of sports industry agglomeration.

Secondly, the health of residents is influenced by various factors, including income^4^, industrial structure^5^, popularization of medical insurance^6^, and environment^7–9^. There is a non-linear relationship between income level and resident health, and uneven income distribution can affect the health status of residents in the region^10,11^. The popularization of medical insurance has a significant positive impact on the health of residents^6^, which can not only reduce the incidence of emergency cases^12^ but also suppress the mortality rates of infants and older adults^13,14^.

Among the factors affecting the health of residents, sports have a significant impact on their health. Warburton et al.^15^ found that the relationship between physical activity and health outcomes is usually curved. Therefore, relatively fewer physical activities can result in significant health benefits. The settings-based health promotion approach forms the basis for the HPSC concept and is first introduced. After that, both obligating and prospecting factors justify the importance of sports clubs addressing health promotion^16^. Moeijes et al.^17^ think that physical activity is associated with health-related quality of life in children (HRQoL). Malm et al.^18^ think physical activity and exercise have significant positive effects in preventing or alleviating mental illness, including depressive symptoms and anxiety or stress-related disease. Hongchuan et al.^19^ found that the health effects of sports consumption on the population aged 35 and above will significantly increase with age, with the most prominent impact on the elderly population aged 65 and above.

Previous studies have shown that industrial agglomeration has a positive impact on economic growth^20–22^, and the sports industry, as one of the driving forces of economic growth, can promote economic growth by driving household consumption and exports^23,24^.On this basis, some scholars have found that the aggregation of the sports industry is conducive to promoting regional economic growth through resource sharing^25^, generating economies of scale and technology spillover effects, and regional economic growth will also attract sports-related industries to gather, leading to an increase in the concentration of the sports industry^26^.

In summary, the existing research mainly focuses on the two directions of sports and residents' health but lacks the impact of sports industry agglomeration on Residents' health. For the effect of the sports industry, scholars have chosen different research regions, research times, and methods to get different research results. It also proves from the side that the development of the regional sports industry differs significantly; the degree of aggregation is low. And it isn't easy to reach a unified result.

## Spatial correlation test of sports industry clustering degree

There are many methods to measure the aggregation degree of the sports industry, such as industry concentration index (CR index), spatial Gini coefficient (G coefficient), location entropy (LQ value), Ellison Glaeser aggregation index (EG index), etc.Given that the scale of the sports industry varies among different regions in China and there are significant differences, it may lead to a lower value-added sports industry in certain areas but higher agglomeration effects in the sports industry. Therefore, this study draws on the method of Weiwu et al.^27^ and uses the Industry Proportion Index (CI index) to evaluate the level of inter-provincial sports industry agglomeration, which compares the regional sports industry value-added with the national sports industry value-added in a particular year. The incomplete statistics and untimely data release of the added value of the sports industry by region are standard, especially before 2010 when there were many missing values in this data. Therefore, this study uses the sum of the cultural, sports, and entertainment industries in the tertiary industry and the manufacturing industries of cultural, artistic, sports, and entertainment products as a substitute indicator for the added value of the sports industry.

Before considering whether to include spatial factors in the model, it is necessary first to determine whether there is spatial autocorrelation. This paper uses the Moran index to test China's spatial correlation of sports industry agglomeration. A geographic weight matrix is a matrix that describes geographic spatial relationships, typically based on spatial distance or geographic distance. It can represent geographical proximity or distance, such as the distance between two locations or the similarity between them. Geographic matrices are crucial in spatial analysis as they help capture spatial correlations. On the one hand, in spatial analysis, geographic matrices are often chosen to capture spatial correlations because they more directly reflect the distance or proximity between geographic locations, making them more suitable for revealing spatial effects. On the other hand, the explanatory variable of this article is the degree of aggregation of the sports industry. The foundation of the sports industry is sports facilities, and fixed-location sports facilities are more dependent on similar geographical locations. Therefore, this article uses a geographic weight matrix for Moran index testing.

The global Moran index reflects the overall characteristics of research variables' degree of spatial correlation. Table 1 shows the global spatial autocorrelation of Moran's I index for the agglomeration degree of the local sports industry in China from 2006 to 2022.

**Table 1:** Spatial correlation test of sports industry agglomeration in China from 2006 to 2022.

Year	Moran’s I	Z(I)	*P* value
2006	0.120*	1.512	0.065
2007	0.111*	1.440	0.075
2008	0.110*	1.452	0.073
2009	0.088*	1.258	0.104
2010	0.112*	1.474	0.070
2011	0.114*	1.500	0.067
2012	0.084*	1.228	0.110
2013	0.227***	2.495	0.006
2014	0.256***	2.729	0.003
2015	0.296***	3.075	0.001
2016	0.305***	3.150	0.001
2017	0.263***	2.820	0.002
2018	0.269***	2.866	0.002
2019	0.225***	2.520	0.006
2020	0.106	1.238	0.108
2021	0.072	0.939	0.174
2022	0.033	0.601	0.274

(1) Z(I) indicates that the new variable is multiple standard deviations under the standard normal distribution. The P-value is obtained according to the significance test method.

(2) ***, ** and * indicate significance at the 1%, 5% and 10% levels, respectively. The table below is the same.

According to Table 1, the spatial correlation of the agglomeration degree of the sports industry in Chinese provinces has a significant positive spatial correlation. Except for 2009, 2012, and 2020–2022, the correlation of China's sports industry agglomeration in other years is substantial, passing at least a 10% significance test. It shows that the regional sports industry agglomeration is not randomly distributed in space but has a significant spatial correlation. The development of the regional sports industry has a substantial relationship with consumption structure and natural factor endowment. China has a vast territory and abundant resources. Geographically, similar regions have similar consumption structures and industrial structures. It results in a significant positive spatial spillover effect in the degree of sports industry agglomeration.

**Figure 1:**
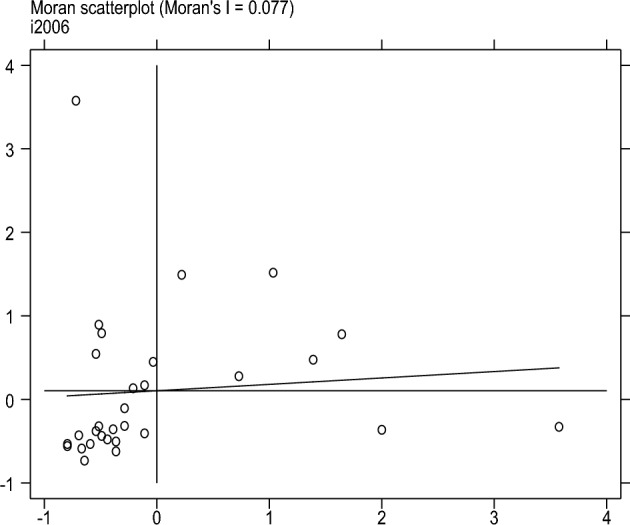
Moran scatter plot from 2006.

**Figure 2:**
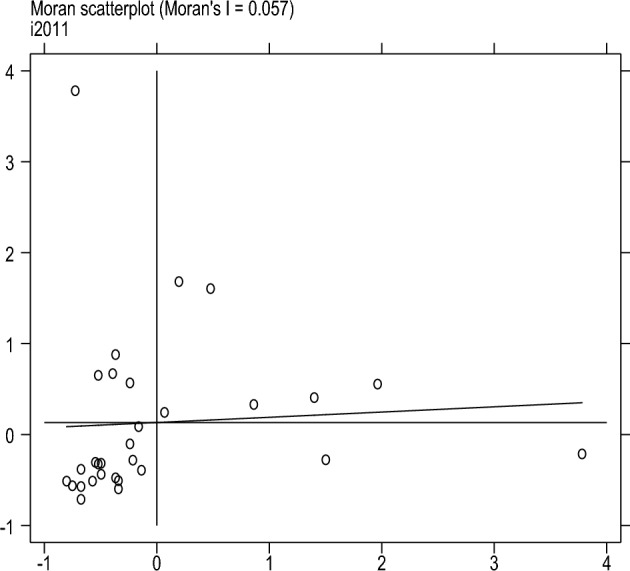
Moran scatter plot from 2011.

**Figure 3:**
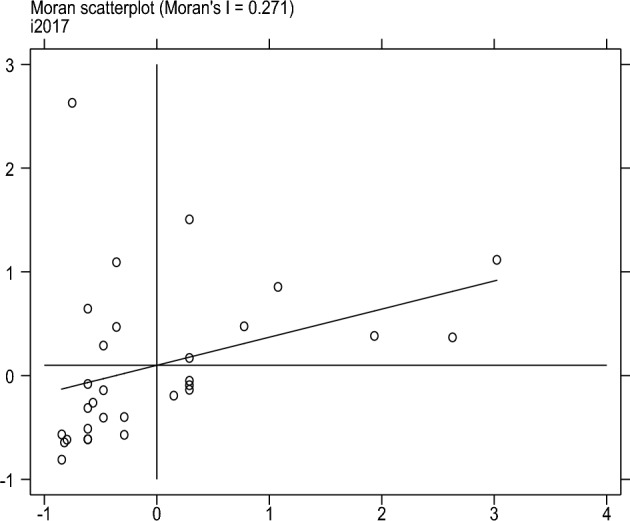
Moran scatter plot from 2017.

**Figure 4:**
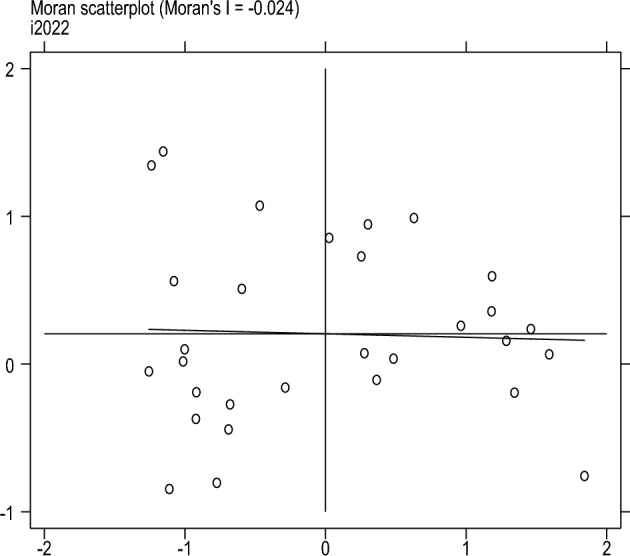
Moran scatter plot from 2022.

This article uses four scatter plots to conduct local spatial autocorrelation tests on the degree of sports industry agglomeration to measure further the spatial correlation and spatial differences between provinces and other cities. Locally Moran’s I, scatter plots can represent the degree of spatial differences and spatial distribution patterns. Figures 1, 2, 3 and 4 shows the Moran’s I scatter plot of the degree of aggregation of China's sports industry in 2006, 2011, 2017, and 2022. The distribution of points in different regions varies significantly in other years, with a relatively average linear fit in 2017. In 2006, more areas were clustered in the third quadrant LL (low-low clustering), followed by the first quadrant HH (high-high clustering). By 2022, the distribution of sports industry clusteringis relatively scattered compared to other years.

In summary, there is spatial autocorrelation and spatial heterogeneity in the clustering level of China's sports industry. The spatial distribution is uneven, and different regions have formed relatively stable spatial patterns.

## Model setting and empirical analysis

### Model setting and descriptive statistical analysis

This article mainly measures spatial effects, where spatial correlation primarily manifests in two aspects: the error term and the lag term of the dependent variable. Before conducting spatial effects regression, the LM test was used, and it was found that the statistical measures were significant at the 1% level, indicating the rationality of choosing a spatial econometric model. The LR test statistic and Wald test statistic are both significant at the 5% level, firmly rejecting the null hypothesis, indicating that the Spatial Durbin (SDM) model cannot degenerate into a Spatial Lag Model (SAR) model or Spatial Error Model (SEM) model. Compared with SEM and SAR models, choosing the SDM model is better. The SDM is a combined extension of the SAR and the SEM, which can be established by adding corresponding constraints to SAR and SEM. Its regression form is:

1 $$ y = X\upbeta + WX{\updelta  + \varepsilon } $$

2 $$ y =\uplambda Wy + X\upbeta + WX\updelta { + }\upvarepsilon $$

In Eqs. (1) and (2), $WX\updelta$ represents the influence of independent variables from adjacent provinces, $\updelta$ represents the coefficient vector corresponding to the observed values of other independent variables in a particular province, $\uplambda$ represents the spatial autoregressive coefficient used to measure the influence of independent variables in this province on the observed values, and $\upvarepsilon$ is the vector of random error terms. Next, this section lists the explanatory variables, dependent variables, and selected control variables one by one.

1. Explained variables: the number of visits per capita ($vpc$). Self-evaluation is the primary measure of residents' health level in academic circles. However, this method contains more personal subjective factors and is not objective and data-based. Therefore, this paper uses macro-level data to indicate residents' health levels. In addition, the health level includes not only physical health but also mental health. Whether the average life expectancy has increased and the mortality rate has decreased is not sufficient to fully measure the health level of the residents. Considering these factors, the mortality rate represents the quantitative dimension of residents' health level, measured in terms of the dead population / the total population at the end of the year.
2. Core explanatory variable: sports industry agglomeration ($SI$). As a comprehensive indicator, measuring the degree of sports industry agglomeration is relatively complex. In the previous section, this paper uses the entropy weight method to calculate the agglomeration degree of the inter-provincial sports industry in China from 2006 to 2022 from the three aspects of the scale, speed, and quality of sports industry agglomeration. The detailed calculation results are presented in the previous section, which will not be repeated here.
3. Control variables

Urbanization level ($urb$): The urbanization level represents the city's civilization and socialization and influences residents' health level from population structure, labor mobility, and social security. This paper uses the proportion of the urban population to the total population at the end of the year to represent the urbanization level.

Per capita GDP ($gdp$): China's economic development is unbalanced. The tertiary industry in economically developed regions develops better than in less developed areas. Per capita GDP can represent the level of regional economic development.

Consumer price index ($cpi$): Residents' consumption level can influence residents' health from the aspects of diet structure and consumption habits. The CPI index can reflect the relative number of price change trends and the degree of consumer goods and services purchased by urban and rural residents in a certain period.CPI can describe the consumption level of residents well.

Per capita park green area ($ppg$): The government's investment in the environment and greenery represents the government's attention to the health level of residents. This paper uses the per capita parkland area to define. The specific estimation method is the area of regional parkland area/total population at the end of the year.

Education investment ($edu$): The emphasis on health can be influenced by the education level of residents and also related to the regional investment in education. There is a lag in the effect of residents' education level on Residents' health levels. Therefore, this paper uses the strength of investment in education to express. The specific measurement method is education budget expenditure / total fiscal budget expenditure.

Aging ($old$): The regional aging rate can represent the age structure, and to a large extent, it directly affects the residents' mortality rate and the number of visits per inhabitant. This paper continues the division method of the China Statistical Yearbook and refers to the population over 65 years old as the elderly population. Aging is measured as the number of aging population / total population.

In the data used in the empirical test, the core explanatory variable is the sports industry's aggregation degree, as measured in the previous section. The explanatory variables were the number of visits per capita. The data are from the China Health Statistics Yearbook (calendar). The data of control variables mainly come from the China Statistical Yearbook, China Industrial Statistical Yearbook, China Urban Statistical Yearbook, the website of the National Bureau of Statistics, and the China Economic and social development statistical database in the previous year. Some indicators, such as urbanization and aging, are calculated based on statistical data. Some of the missing data are supplemented from the statistical yearbooks of provinces and municipalities, and some of the missing data are complemented by linear interpolation and linear trend methods. In addition, considering the unique characteristics and data availability of the four regions of Tibet Autonomous Region, Hong Kong Special Administrative Region, Macao Special Administrative Region, and Taiwan, the data of these four regions were excluded from the data collation process. Finally, the balance panel data were obtained for 30 provinces from 2006 to 2022. To eliminate the influence of heteroscedasticity and quantity stiffness of related variables on the results, the per capita GDP is logarithmicized in this paper. Table 2 reports the descriptive statistical analysis of all variables.

**Table 2:** Descriptive statistical analysis of variables.

	Variable name	Variable symbols	Minimum value	Maximum value	Mean value	Standard deviation
Explanatory variables	Aggregation degree in the sports industry	${\text{SI}}$	0.0010	0.197	0.0327	0.0361
Explained variable	Number of visits per capita	$vpc$	0.430	11.65	2.982	2.292
Control variable	Urbanization	$urb$	27.46	89.6	56.81	13.75
	Per capita GDP	$gdp$	8.663	12.15	10.64	0.6283
	Consumer price index	$cpi$	97.65	102.8	108.00	70.75
	Per capita park green area	$ppg$	2.53	20.38	10.19	3.755
	Investment intensity of education funds	$edu$	0.1	1.19	0.2074	0.1762
	Aging	$old$	0.080	15.16	7.456	4.416

### Spatial Durbin regression results

The Hausman test will be performed under the geographical weight matrix. The P-value of the Hausman test result is 0.0012, indicating that using the time–space double fixed effects model is more effective when choosing the SDM model. Based on this, this article analyzes the spatial Durbin model with time–space dual fixed effects. Model (1) represents the spatial Durbin regression results, while models (4), (5), and (6) represent the direct, indirect, and total effects of variables, respectively.

From Table 3, it can be seen that the core explanatory variable, the degree of aggregation of the sports industry, has passed the significance tests of direct effect, indirect effect, and total effect at the 1% level, and the spatial regression coefficient is − 6.783, indicating that the degree of aggregation of the sports industry can reduce the number of per capita visits and has a positive spatial spillover effect on resident health. It not only promotes the health level of residents in the province but also has a spatial spillover effect on surrounding areas.

**Table 3:** Spatial panel estimation results under geographical weight matrix.

Variable	(1)	(2)	(3)	(4)	(5)	(6)
	Main	Spatial	Variance	LR-Direct	LR-Indirect	LR-Total
SI	− 6.783*** (− 3.97)			− 7.015*** (− 4.00)	− 17.54*** (− 5.31)	− 24.55*** (− 6.15)
urb	0.0014(0.12)			0.0023 (0.835)	0.0869*** (5.94)	0.0892*** (5.46)
lngdp	1.392*** (8.24)			1.405*** (8.57)	− 0.2606** (− 2.19)	1.144*** (5.13)
cpi	0.0003(0.38)			0.0003 (0.698)	− 0.0005(− 0.22)	− 0.0003 (− 0.14)
ppg	− 0.0851***(− 5.23)			− 0.0877*** (− 5.65)	− 0.1077***(− 5.32)	− 0.1954***(− 8.48)
edu	− 0.3124(− 0.98)			− 0.2996 (− 0.98)	0.2022 (0.87)	− 0.0974 (− 0.46)
old	− 0.1522** (− 8.40)			− 0.1503*** (− 8.36)	0.0628*** (4.62)	− 0.0874*** (− 4.90)
Constant term	− 10.29***(− 7.44)					
${\text{rho}}$		28.23*** (15.41)				
Sifma2_e			0.3254*** (15.40)			
Observations	510	510	510	510	510	510
R-squared	0.8942	0.8942	0.8942	0.8942	0.8942	0.8942
Number of regions	30	30	30	30	30	30

* * *, * *, and * represent significant at 1%, 5% and 10% respectively.

This empirical result is consistent with reality: firstly, the gathering of the sports industry is usually accompanied by various sports events, fitness activities, and the construction of sports facilities. These activities and facilities will enhance the health awareness of local and surrounding residents, encourage them to participate in sports exercise more actively, and thus reduce the incidence of diseases. The construction of large sports venues, fitness clubs, parks, and other fitness facilities makes it easier for residents in the surrounding areas to access various forms of exercise and have more choices. The services provided by these facilities can meet residents' fitness needs and reduce the incidence of chronic diseases. Secondly, regions with high levels of sports industry aggregation often have higher levels of economic development and pay more attention to living environment and community construction, such as greening and leisure facilities. An improved living environment and a positive community atmosphere are beneficial for residents and surrounding residents to maintain a healthy lifestyle and reduce the occurrence of diseases. Finally, sports activities are not only helpful to the body but also have a positive effect on mental health. Participating in sports can release stress, enhance happiness, reduce psychological problems such as anxiety and depression, and thus reduce the need for medical treatment caused by mental health issues. Regions with high levels of sports industry aggregation can guide residents to participate in sports activities and attract residents from surrounding areas to engage in sports activities.

### Robustness test

Many factors influence the health level of residents. Changes in the economic environment, development stage, and residents' consumption habits will cause fluctuations. To ensure the preciseness of the empirical study, the article tests the robustness of the spatial Durbin model regression results by changing explanatory variables and spatial weight matrices, and it conducts a simple analysis.

#### Replacement of explanatory variables

Sports, culture, and entertainment belong to the tertiary industry, so the industrial structure ($is$) can also represent the aggregation of the sports industry in a region to a certain extent. To test the robustness of the above empirical model, this paper takes the industrial structure of different provinces from 2006 to 2022 as the explanatory variable. At present, there are many measurement indicators for upgrading industrial structures. Generally speaking, upgrading industrial structure can be characterized by the relative scale changes among industries. This paper uses the ratio of the tertiary industry's added value to the secondary industry's added value to measure the upgrading of the industrial structure.

#### Replacement spatial weight matrix

The spatial Durbin model used in the previous text is a geographic weight matrix, which has been replaced with an economic weight matrix to test the robustness of empirical results. The economic weight matrix is used to measure the relative importance of different economic indicators or factors in the overall economy, which can, to some extent, reflect the spatial spillover effect of the degree of sports industry aggregation.

Table 4 shows the robustness test results. Based on the regression results using industrial structure as the explanatory variable, the regression coefficient of industrial structure in Model (1) is − 0.1075, which is significant at the 10% level, consistent with the results in Table 4. Model (2) shows the spatial Durbin regression results replaced by an economic weight matrix with a regression coefficient of − 6.812, passing the 1% significance test. The regression results for the aggregation degree of the sports industry in Table 3 are consistent. Therefore, the above empirical results are robust.

**Table 4:** Robustness test.

	Replacement of explanatory variables	Replacement spatial weight matrix
	(1)	(2)
SI		− 6.812*** (− 3.64)
Industrial structure	− 0.1075* (− 1.88)	
Control variable	Yes	Yes
Constant term	3.222*** (2.78)	− 14.14*** (− 9.73)
Sifma2_e	0.1982*** (15.24)	0.3910*** (15.42)
Observations	510	510

## Conclusions, recommendations, and research outlook

### Conclusion

With China's economic development, medical and healthcare expenditures have increased in all regions, and the average life expectancy of residents has risen dramatically. However, the health level of residents, especially the health level of adolescents, has remained low for a long time.

This article measures the degree of sports industry agglomeration in different regions of China, uses the Moran index to verify the global autocorrelation of sports aggregation, and then uses the spatial Durbin model to verify the spatial spillover effect of sports industry agglomeration on residents' health levels. The following conclusions are drawn: firstly, there is spatial autocorrelation and spatial heterogeneity in the level of sports industry agglomeration in China. The spatial distribution is extremely uneven, and different regions have formed relatively stable spatial patterns. Secondly, the degree of aggregation of the sports industry can reduce the number of per capita visits, which has a positive spatial spillover effect on the health of residents. It can not only promote the health level of residents in the province but also have a spatial spillover effect on surrounding areas.

### Policy recommendations

With the successful introduction of the Chinese government's policies to develop the sports industry, the sports industry is in a golden period of development. From the above research results, it can be seen that sports industry agglomeration can significantly improve the health level of residents and reduce the mortality rate and the number of visits per capita. The scientific layout and planning of the spatial pattern of the sports industry ensure the efficient utilization of resources and the industry's sustainable development. To promote the sports industry's gathering and improve residents' health levels. According to the research conclusion, this paper puts forward the following three suggestions:

First, investment should be increased in the training of sports talents. The sports industry involves sports, economics, management, and other fields. Therefore, the importance of sports talents in developing the sports industry is self-evident. Historical reasons exist for the current neglect of sports-related personnel training in China. There has always been a mainstream idea in China that "everything is inferior; only reading is superior."After the founding of new China, the value of sports and other related talents was recognized, which will inevitably affect the cultivation of sports talents. Because of this problem, this paper puts forward the following suggestions for training sports talents. In response to this problem, this paper makes the following suggestions for cultivating sports talents. First, we can jointly train sports talents with universities, training institutions, and enterprises. Colleges and universities should give full play to the advantages of their educational resources and, through cooperation with sports enterprises, set up comprehensive majors in the sports industry to cultivate comprehensive talents with specific management knowledge and sports knowledge. Secondly, vigorously introduce sports-related talents. At present, the talent introduction policies of most local governments still have significant limitations. It is suggested that the restrictions on the introduction of talent should be liberalized, and the introduction of sports talents should be vigorously promoted to promote the development of the regional sports industry. Finally, we should intensify efforts to encourage innovation discipline and promote the theoretical construction of the sports industry. In the final analysis, talent training still depends on theoretical construction. Sounding the theoretical foundation and encouraging innovation is necessary to cultivate comprehensive sports talents.

Second, accelerate the construction of a sports center city. A sports center city is a city with a significant influence on sports ecology, sports affairs, and sports industry within a specific region. The construction of a sports center city should focus on its advantages of high industrial relevance, intense radiation, and high added value and comprehensively promote the regional sports industry's development with a point. It will also expand the construction of stadiums and reduce the cost of physical activity for residents, thus improving their health. However, it should also be noted that when the sports industry is excessively concentrated, reducing the inhibitory effect of the efficiency spillover effect on the industrial efficiency of the surrounding areas is essential.

Third, strengthen the residents' attention to sports." A robust youth makes a strong nation." A healthy body for young people is the foundation for the sustainable and stable development of the country. It is far from enough to rely solely on the national construction of the sports industry. Sports culture can only truly realize its value when deeply rooted in the hearts and minds of the people. The government should strengthen the publicity of physical exercise and formulate compulsory measures to regulate the physical exercise of teenagers in the compulsory education stage. In addition, introducing some sports events suitable for full participation can also effectively improve residents' enthusiasm for physical exercise—large-scale sports events such as the Winter Olympics in Beijing in 2022. Small-scale sporting events such as marathons are held in many cities. It can guide citizens to participate in mass sports and form a positive and healthy mass sports culture.

### Theoretical contributions

Human Capital Theory holds that human capital is the sum of knowledge, skills, and experiences that individuals possess through education, training, and other means. Increasing investment in sports talents can be seen as an investment in human capital, thereby improving the productivity and competitiveness of the sports industry. By increasing investment in sports talent cultivation, the government can promote a more effective allocation of resources in the sports industry, thereby improving overall efficiency. Cultivating sports composite talents can promote optimizing and upgrading the sports industry structure, enhancing the industry's competitiveness and innovation ability.

Urban economics theory believes that cities can become the center of specific fields due to their advantages in resource allocation, industrial accumulation, innovation, etc., thereby promoting the development of related industries and the economy. Building a sports center city helps to encourage the development of the sports industry in the region and drives economic growth in surrounding areas. The construction of a sports center city helps determine the city's functional positioning and enhance its brand image and attractiveness.

Social psychology theory emphasizes the role of social culture in shaping individual behavior and attitudes. Increasing the attention of residents to sports to promote the development of healthy lifestyles within the framework of cultural inheritance and social identity. Increasing residents' attention to sports can help improve the overall health level of society, thereby reducing medical costs improving productivity and quality of life. Through the in-depth promotion of sports culture and the popularization of physical exercise, social cohesion can be enhanced, and social harmony and stability can be promoted.

### Research outlook

This paper empirically tests the relationship between regional sports industry agglomeration and residents' health levels based on qualitative analysis. However, due to the availability and applicability of data, this paper only uses provincial-level data from 2006 to 2022. Due to the small sample size, the research in this paper has certain limitations in depth and breadth. In future research, we will continue to pay attention to the development of China's sports industry. We try our best to find the data at the prefecture level and city level and study the heterogeneity of the relationship between sports industry concentration and residents' health level from two aspects of urban and rural areas.

On the other hand, research on the health level of Chinese residents has been carried out from the micro level, such as using household micro-adjustment data to study its influencing factors. However, there is a lack of correlation analysis with sports industry agglomeration. Therefore, the following research can consider exploring the impact of residents' sports-related consumption structure on health levels from a micro perspective. It even combines the macro research results with the micro research results. It combines China's experience with international experience to provide different paths to improve the health level of residents in China.

## Data Availability

The datasets used and analyzed during the current study are available from the corresponding author upon reasonable request.
